# CXCR4-Directed PET/CT in Patients with Newly Diagnosed Neuroendocrine Carcinomas

**DOI:** 10.3390/diagnostics11040605

**Published:** 2021-03-29

**Authors:** Alexander Weich, Rudolf A. Werner, Andreas K. Buck, Philipp E. Hartrampf, Sebastian E. Serfling, Michael Scheurlen, Hans-Jürgen Wester, Alexander Meining, Stefan Kircher, Takahiro Higuchi, Martin G. Pomper, Steven P. Rowe, Constantin Lapa, Malte Kircher

**Affiliations:** 1 Department of Internal Medicine I, Gastroenterology, University Hospital Würzburg, 97080 Würzburg, Germany; weich_a@ukw.de (A.W.); Scheurlen_m@ukw.de (M.S.); meining_a@ukw.de (A.M.); 2 European Neuroendocrine Tumor Society (ENETS) Center of Excellence, NET Zentrum, University Hospital Würzburg, 97080 Würzburg, Germany; werner_r1@ukw.de (R.A.W.); Buck_A@ukw.de (A.K.B.); Constantin.Lapa@uk-augsburg.de (C.L.); 3 Department of Nuclear Medicine, University Hospital Würzburg, 97080 Würzburg, Germany; hartrampf_p@ukw.de (P.E.H.); serfling_s1@ukw.de (S.E.S.); higuchi_t@ukw.de (T.H.); 4 The Russell H. Morgan Department of Radiology and Radiological Science, Division of Nuclear Medicine and Molecular Imaging, Johns Hopkins University School of Medicine, Baltimore, MD 21218, USA; mpomper@jhmi.edu (M.G.P.); srowe8@jhmi.edu (S.P.R.); 5 Pharmaceutical Radiochemistry, Technische Universität München, 80333 München, Germany; h.j.wester@tum.de; 6 Institute of Pathology, University of Würzburg, 97080 Würzburg, Germany; stefan.kircher@uni-wuerzburg.de; 7 Graduate School of Medicine, Dentistry and Pharmaceutical Sciences, Okayama University, Okayama 700-8558, Japan; 8 Comprehensive Heart Failure Center, University Hospital Würzburg, 97080 Würzburg, Germany; 9 Nuclear Medicine, Medical Faculty, University of Augsburg, 86156 Augsburg, Germany

**Keywords:** CXCR4, NET, NEC, ^68^Ga-Pentixafor, ^18^F-FDG

## Abstract

We aimed to elucidate the diagnostic potential of the C-X-C motif chemokine receptor 4 (CXCR4)-directed positron emission tomography (PET) tracer ^68^Ga-Pentixafor in patients with poorly differentiated neuroendocrine carcinomas (NEC), relative to the established reference standard ^18^F-FDG PET/computed tomography (CT). In our database, we retrospectively identified 11 treatment-naïve patients with histologically proven NEC, who underwent ^18^F-FDG and CXCR4-directed PET/CT for staging and therapy planning. The images were analyzed on a per-patient and per-lesion basis and compared to immunohistochemical staining (IHC) of CXCR4 from PET-guided biopsies. ^68^Ga-Pentixafor visualized tumor lesions in 10/11 subjects, while^18^F-FDG revealed sites of disease in all 11 patients. Although weak to moderate CXCR4 expression could be corroborated by IHC in 10/11 cases, ^18^F-FDG PET/CT detected significantly more tumor lesions (102 vs. 42; total lesions, *n* = 107; *p* < 0.001). Semi-quantitative analysis revealed markedly higher ^18^F-FDG uptake as compared to ^68^Ga-Pentixafor (maximum and mean standardized uptake values (SUV) and tumor-to-background ratios (TBR) of cancerous lesions, SUV_max_: 12.8 ± 9.8 vs. 5.2 ± 3.7; SUV_mean_: 7.4 ± 5.4 vs. 3.1 ± 3.2, *p* < 0.001; and, TBR 7.2 ± 7.9 vs. 3.4 ± 3.0, *p* < 0.001). Non-invasive imaging of CXCR4 expression in NEC is inferior to the reference standard ^18^F-FDG PET/CT.

## 1. Introduction

Neuroendocrine neoplasms (NEN) of the gastro-entero-pancreatic tract (GEP-NEN) are histologically classified into well differentiated neoplasia (G1, G2, G3) and poorly differentiated carcinomas (NEC) based on morphological features, mitotic count, and proliferation index (Ki-67) [1]. This established grading scheme is essential for guiding the treating physician towards an effective therapy that includes “cold” somatostatin analogues (SSA) and radiolabelled “hot” SSAs that target somatostatin receptors (SSTR) on the tumor cell surface [2]. Such sophisticated approaches of receptor interaction specific for neuroendocrine tumor cells have led to significant gains in health-related quality of life, as well as markedly prolonged progression-free and overall survival in low and intermediate grade NEN [3,4,5]. 

However, patients suffering from neuroendocrine carcinomas (NEC), which are characterized by a considerably more aggressive tumor growth, do not benefit from cold or hot SSAs, which can be attributed to tumor dedifferentiation with a loss of SSTR expression [2]. In fact, in 2017, the World Health Organization changed its classification of NEN to better account for the fact that well differentiated G3 NEN and poorly differentiated NEC are genetically two different diseases with very divergent clinical outcomes [6]. Historically high grade NEN have been treated with chemotherapy, but, more recently, several studies have shown that peptide receptor radiotherapy is effective not only in G1/G2, but also in G3 NEN [7,8,9]. However, NEC cannot be treated the same way and therapy options for these highly aggressive malignancies are very limited and usually include chemotherapy regimens with limited efficacy [10]. Therefore, novel treatment options in poorly differentiated NEC are urgently needed [10].

From an imaging perspective, focus in high grade NEN and NEC lies in capturing all SSTR-negative tumor lesions. Because an increase in tumor aggressiveness is tightly linked to an elevated glycolytic activity, ^18^F-fluorodeoxyglucose (^18^F-FDG) positron emission tomography/computed tomography (PET/CT) has been more widely used for staging G3 NEN in recent years, and it has also found its way into the clinical practice guidelines [11].

One alternative to ^18^F-FDG might be the targeting of receptors other than the SSTR. In a recent study, it was shown that an overexpression of the C-X-C motif chemokine receptor 4 (CXCR4) in NEN is associated with a more aggressive and dedifferentiated tumor phenotype, which is also accompanied by a decrease in SSTR expression [12]. A subsequent study investigating the potential of CXCR4-directed imaging using the novel PET tracer ^68^Ga-Pentixafor confirmed an overexpression of CXCR4 in higher grade NEN and poorly differentiated NEC [13].

Of note, ^68^Ga-Pentixafor has a theranostic “twin”, called Pentixather, which can be labelled with the beta-emitters lutetium-177 or yttrium-90 for CXCR4-directed endoradiotherapy (ERT), and it has been used with varying degrees of success in multiple myeloma and other haemato-oncological diseases [14,15,16]. In theory, CXCR4-directed ERT might offer another treatment option for advanced NEC patients, in a similar manner as peptide receptor radiotherapy is used in NEN. In this setting, ^68^Ga-Pentixafor PET/CT would serve as a non-invasive measure of sufficiently high CXCR4 expression in all tumor lesions to allow for an evaluation of CXCR4-directed ERT [13,14,15,16].

In the present study, we examined the diagnostic potential of CXCR4-directed imaging in NEC, and compared its performance to the reference standard ^18^F-FDG PET/CT. Additionally, we investigated whether the imaging and biopsy results might serve as fundament for the potential of CXCR4-targeted ERT.

## 2. Materials and Methods

### 2.1. Patients

We searched our PET database from November 2015 until October 2018 and included patients with newly diagnosed NEC, who underwent PET/CT imaging with ^68^Ga-Pentixafor and ^18^F-FDG. Out of 1,134 patients, 11 (0.97%) subjects (nine males, two females) met eligibility criteria. The mean age was 65 ± 12 years (range, 45–80). The primary tumor was located in the stomach (*n* = 3), pancreas (*n* = 2), oesophagus (*n* = 2), ileum (*n* = 1), and the rectum (*n* = 1). In the remaining subjects, no primary tumor could be identified (*n* = 2).

PET-guided biopsies were taken of lesions with discrepant tracer uptake to verify advanced dedifferentiation, and the specimens were examined for CXCR4 expression. Of these biopsies, 45.5% were taken from the primary tumor (*n* = 5), 18.2% from lymph node metastases (*n* = 2), and 36.3% from haematogenic metastases (*n* = 4), respectively. All of the patients signed informed consent, and this study has been approved by the local ethical board (IRB approval: 2016100701; date of approval: 12.10.2016).

Parts of this cohort have been analyzed in [13]. Table 1 provides comprehensive patients’ characteristics, as well as an overview of PET and biopsy results.

### 2.2. PET/CT Imaging

^68^Ga-Pentixafor and ^18^F-FDG were synthesized in-house with a 16 MeV Cyclotron (GE PETtrace 6; GE Healthcare, Milwaukee, WI, USA). For synthesis of ^68^Ga-Pentixafor, a fully automated, GMP-compliant method using a GRP module (Scintomics, Fürstenfeldbruck, Germany) that was equipped with disposable single-use cassette kits (ABX, Radeberg, Germany) was used, as described in [17]. ^18^F-FDG was synthesized in accordance with the manufacturer’s instructions (GE FASTlab, Chicaco, IL, USA). Prior to administration of these radiotracers, radiochemicals were analyzed by high performance liquid chromatography for radiochemical identity and purity. Quality control of ^68^Ga-Pentixafor was conducted according to the standards, as outlined in European Pharmacopoeia for ^68^Ga-edotreotide (European Pharmacopoeia; Monograph 01/2013:2482; available at www.edqm.eu (accessed on 1 December 2020)).

CXCR4-directed and ^18^F-FDG PET/CT scans were performed on a dedicated PET/CT scanner (Siemens Biograph mCT 64; Siemens Medical Solutions, Erlangen, Germany), in the case of FDG after a 6-h fasting period to ensure serum glucose levels below 130 mg/dL, in case of ^68^Ga-Pentixafor without any special patient preparation. The injected activities for ^68^Ga-Pentixafor were 115 ± 30 MBq (range, 72–164) and 303 ± 12 MBq (range, 294–330) for ^18^F-FDG, respectively. Mean delay between scans was eight days (range, 1–23 days). Whole-body (top of the skull to knees) PET scans were performed one hour after administration of the radiopharmaceutical. In CXCR4-directed PET, corresponding low-dose CT scans for attenuation correction and anatomical correlation were subsequently acquired (35 mAs, 120 keV, a 512 × 512 matrix, 5 mm slice thickness, increment of 30 mm/s, rotation time of 0.5 s, and pitch index of 0.8). In the case of FDG PET a monophasic, contrast-enhanced CT (CARE Dose 4D, 160 mAs, 120 kV, 512 × 512 matrix, 5 mm slice thickness, slice collimation 64 × 0.6 mm, pitch index 1.4) was acquired. The PET images were reconstructed using standard parameters (HD-PET, 3 iterations, 24 subsets, Gaussian filtering: 2 mm, resolution: axial resolution: 5 mm, in-plane resolution: 4 × 4 mm^2^) with corrections for attenuation (CT-based), dead-time, random events, and scatter.

### 2.3. Image Analysis

PET/CTs were separately analyzed by two experienced investigators (CL and MK) that were blinded to the respective other PET scan as well as to all clinical information. Lesions were visually determined as focally increased tracer retention when compared to surrounding normal tissue or contralateral structures. Images were first inspected visually. Subsequently, the maximum and mean standardized uptake value (SUV_max_ and SUV_mean_) of all potential lesions was derived by placing volumes of interest (VOI) of 10 mm diameter or more around them and then applying an isocontour of 40%. To normalize uptake and account for background activity, mean blood pool activity was measured by placing a 10 mm VOI in the right atrium. Afterwards, a target-to-background ratio (TBR) was calculated by dividing SUV_max_ (lesion) by SUV_mean_ (blood pool). Analysis of data was performed on both a per-patient and a per-lesion basis. The tumor manifestations with the highest tracer uptake (hottest haematological and lymph node (LN) metastases) in the respective PET scans was used as a comparison parameter in the per-patient analysis. CT was used as reference standard in the per-lesion analysis.

### 2.4. Immunohistochemistry

The biopsies were stained using an anti-CXCR4 rabbit polyclonal antibody (ab2074; Abcam, Cambridge, UK), and detected and visualized using the Dako EnVision-HRP rabbit labeled polymer/DAB. Counterstaining was performed with hematoxylin. CXCR4 positivity of vascular epithelium served as internal and adrenocortical tissue as external positive control. The intensity of CXCR4 expression was visually rated using a four-point scoring scale (0 = absent, 1 = weak, 2 = moderate, 3 = intense). The Ki-67 labeling index after immunostaining for Ki67 (monoclonal, clone Ki-67, 1:50, Dako, Hamburg, Germany) was calculated by determining the number of positive nuclei under 100 tumor cells per high power field (×400) in a total of 10 fields per sample in order to determine the proliferative activity of tumor cells. SUV_mean_/SUV_max_ of the respective biopsied lesion was correlated to the intensity of receptor expression and proliferation activity.

### 2.5. Statistics

Quantitative variables are expressed as mean ± standard deviation (if normally distributed), or as median and range (if not normally distributed). Paired *t*-tests were used to compare uptake (ratios) of ^18^F-FDG and ^68^Ga-Pentixafor in corresponding lesions. Pearson’s correlation coefficients (r) were calculated in order to assess the association between uptake (ratios) of both tracers. A *p*-value of <0.05 was considered to be statistically significant.

## 3. Results

### 3.1. CXCR4-Directed PET/CT Is Inferior to ^18^F-FDG PET/CT in NECs

#### 3.1.1. Per-Patient Analysis

FDG-avid lesions were detected in all patients (*n* = 11) and the primary tumor was visualized in 81.8% of cases (*n* = 9). LN metastases were observed in 72.7% of patients (*n* = 8), most of them occurring locoregionally around the primary tumor (*n* = 7), and only a few of them being distant (*n* = 3). Haematogenic FDG^+^ metastases were found in 81.8% of patients (*n* = 9), predominantly located in the liver (*n* = 9), while the remaining metastases occurred in lung (*n* = 4) and bone (*n* = 2). The mean SUV_max_ of the primary tumor was 13.3 ± 8.5 (range, 5.2–31.9), of the hottest LN metastasis 9.5 ± 5.8 (range, 2.8–21.3) and of the hottest organ metastasis 17.5 ± 12.4 (range, 3.4–40.5) and, respectively (Table 2).

^68^Ga-Pentixafor PET/CT identified CXCR4^+^ lesions in 90.1% of patients (*n* = 10) and detected the primary tumor in 72.7% of cases (*n* = 8). LN metastases were detected in 63.6% of patients (*n* = 7), with most of them occurring locoregionally around the primary tumor (*n* = 7) and only a few of them being distant (*n* = 2). Haematogenic CXCR4^+^ metastases were found in 54.5% of subjects (*n* = 6), being predominantly located in the liver (*n* = 5), with the remaining metastases occurring in bone (*n* = 2), lung (*n* = 2) and other organs (*n* = 1).

The primary tumor had an average SUV_max_ of 7.7 ± 2.2 (range, 3.7–10.6), the hottest LN metastasis of 8.2 ± 2.4 (range, 3.7–11.3), and the hottest haematogenic metastasis of 9.7 ± 4.6 (range, 3.6–15.9), respectively (Table 2).

#### 3.1.2. Per-Lesion Analysis

One-hundred-seven cancerous lesions were analyzed (primary tumor, *n* = 9; LN metastases, *n* = 34; haematogenic metastases, *n* = 64). ^18^F-FDG PET/CT was visually positive in 95.3% (102/107) of lesions, exhibiting a mean SUV_max_ of 12.8 ± 9.8 (range 2.0–40.5), a mean SUV_mean_ of 7.4 ± 5.4 (range 2.0–26.8), and a TBR of 7.2 ± 7.9 (range 1.0–34.4).

^68^Ga-Pentixafor PET/CT was visually positive in 39.3% (42/107) of lesions, exhibiting a mean SUV_max_ of 5.2 ± 3.7 (range 1.0–15.9), a mean SUV_mean_ of 3.1 ± 3.2 (range 0.8–9.4), and a TBR of 3.4 ± 3.0 (range 0.5–9.9).

#### 3.1.3. Comparison of ^18^F-FDG and ^68^Ga-Pentixafor

On a per-person analysis ^18^F-FDG identified more patients with lesions (any), more primary tumors, and more subjects with lymph node and haematogenic metastases, respectively (all *p* = n.s.).

**Figure 1:**
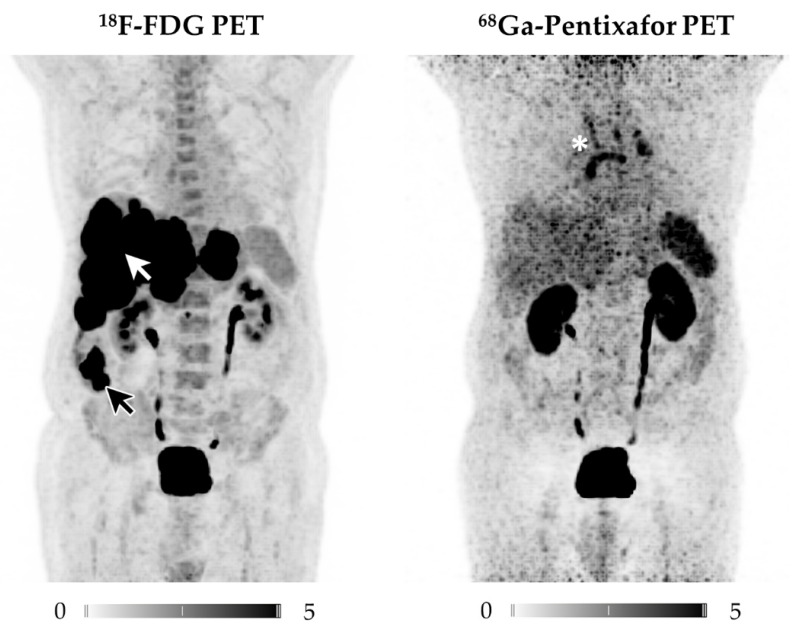
Displayed are Maximum Intensity Projections (MIP) of the ^18^F-FDG (left) and ^68^Ga-Pentixafor PET scans of patient #2. Whereas ^18^F-FDG depicts the ileal primary (black arrow) as well as multiple liver metastases (white arrow), none of the tumor manifestations are revealed by CXCR4-directed PET imaging. Incidental finding: The mediastinal tracer uptake in ^68^Ga-Pentixafor PET (white star) was traceable to enlarged mediastinal lymph nodes, most likely due to chronic lung fibrosis and not related to NEC, as follow-up imaging confirmed.

Analysis on a per-lesion basis revealed the superiority of ^18^F-FDG over ^68^Ga-Pentixafor (102 vs. 42; total lesions, *n* = 107; *p* < 0.001; Figure 1). No correlation was found between tracer uptake in corresponding lesions.

Visually, ^18^F-FDG PET had a far higher tracer uptake as well as tumor-to-background contrast when compared to CXCR4-directed imaging, which could also be semi-quantitatively confirmed (*p* < 0.001).

#### 3.1.4. Immunohistochemistry

**Figure 2:**
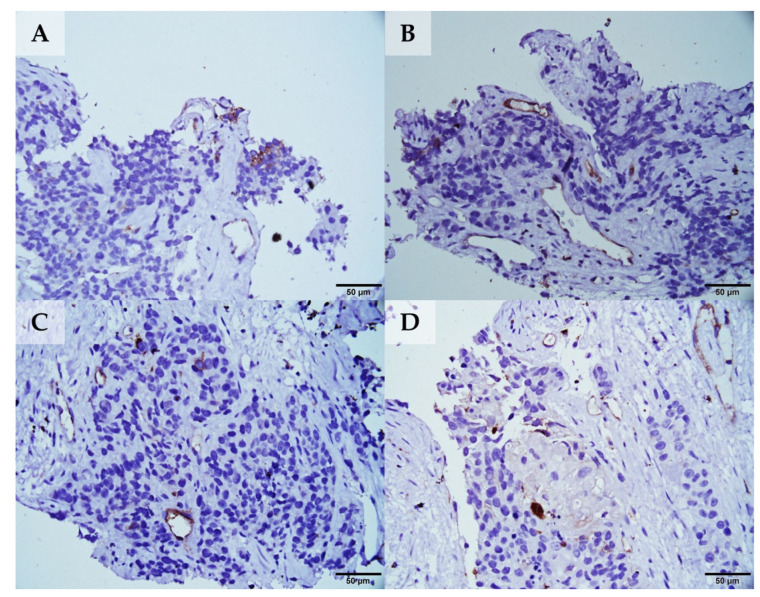
Display of immunohistochemistry of a liver metastasis (**A**–**D**) obtained from patient #2 (also refer to Figure 1). Staining for CXCR4 was rated weak (score of 1) with only single tumor cells (<5%) demonstrating chemokine receptor positivity. Magnification: 600×.

Immunohistochemical staining was able to validate CXCR4 expression in most cases (90.9%; 10/11). The intensity of CXCR4 expression was rated “weak” in 7/11 specimens (Figure 2), “moderate” in 2/11, and “strong” in only 1/11. One biopsy did not provide enough material for CXCR4 staining (patient #11). Mean Ki-67 index of biopsy specimens was 76 ± 11% (range, 45–90).

^68^Ga-Pentixafor uptake of the respective lesion was corroborated by histology in 7/11 subjects; however, there was no correlation between IHC score and ^68^Ga-Pentixafor uptake (*p* = 0.24).

## 4. Discussion

This is the first study to evaluate the performance of CXCR4-directed imaging with the PET tracer ^68^Ga-Pentixafor in a homogenous cohort of treatment-naïve patients suffering from neuroendocrine carcinomas. NEC that do not originate in the lungs are very rare and they are characterized by extremely rapid disease progression. The lack of effective treatment options is one of the biggest challenges in the management of NEC, as most of these carcinomas are metastatic at diagnosis and usually do not respond well to conventional chemotherapy [6].

The overexpression of CXCR4 has been reported in well differentiated, high grade NEN with a strong inverse correlation between grade of differentiation and intensity of receptor expression [12]. This observation was supported by a pilot study that visualized CXCR4 expression in twelve NEN patients (including NEC) using ^68^Ga-Pentixafor PET/CT and concluded that CXCR4-directed imaging might offer diagnostic potential and open the way for CXCR4-directed ERT [13]. In light of these encouraging results, we searched our PET database for a larger cohort of dedifferentiated NEN and identified a substantial, but still rather small, cohort of NEC patients that underwent a dual-tracer imaging protocol at the time of primary diagnosis prior to treatment initiation.

Although ^68^Ga-Pentixafor identified CXCR4^+^ lesions in over 90% of patients and IHC of biopsy samples showed at least weak CXCR4 expression in all specimens, a substantial portion of FDG^+^ findings was missed by CXCR4-targeted imaging (102 vs. 42 detected lesions out of 107 total lesions; *p* < 0.001). These findings validate the results from other studies that reported only moderate tracer accumulation in a variety of solid tumor entities, despite high CXCR4 expression in surgical specimens [18,19]. One reason for this discrepancy might be the intracellular localization of the receptor, as ^68^Ga-Pentixafor only binds to receptors that are expressed on the cell surface, yet IHC routinely detects receptors located in the cytosol.

However, there may be other purposes for CXCR4-directed imaging beyond staging. Initial data suggest that CXCR4 overexpression in NEN is strongly associated with shortened overall survival [12]. Therefore, one may speculate that a non-invasive read-out of CXCR4 expression might serve as a risk stratification tool or help to identify patients prone to early progression. As a result, CXCR4-directed PET/CT prior to initiation of anti-tumor therapy might guide the referring treating physician towards appropriate treatment selection at an early stage of disease progression.

In nuclear oncology, recent years have witnessed an expanded use of CXCR4-targeted ERT in the treatment of multiple myeloma and other hematologic malignancies [14,15,16,20]. The prospect that ^68^Ga-Pentixafor, in addition to disease staging and (potentially) risk stratification, could open avenues for a theranostic approach using the therapeutic counterpart ^177^Lu-Pentixather, is not supported by our data. Especially when considering that CXCR4-directed ERT leads to bone marrow ablation and would require autologous stem cell support [20], the inadequate and heterogenous PET signal suggests that a successful use of this treatment in NEC is very unlikely. However, there is an increasing number of approaches employing ^68^Ga-Pentixafor PET/CT to investigate the in vivo CXCR4 expression in other solid tumors like oesophageal cancer or vestibular schwannoma [21,22]. Moreover, Osl et al. recently introduced a second generation CXCR4 ligand with potentially improved tumor retention [23]. This novel theranostic twin [^68^Ga/^177^Lu]DOTA-r-a-ABA-CPCR4 should be also further evaluated in high grade NET and NEC. One may speculate that, with enhanced ligand internalization, a theranostic approach is more likely, even in high grade NET/NEC.

However, CXCR4-targeted PET/CT could also be employed to trigger targeted therapy assessing the identical or at least comparable target on a (sub)cellular level [24]. For instance, a recent study reported on the combination of the CXCR4-inhibitors AMD3100 and RAD001, which reduced cell growth in human NET cells [25]. Therefore, ^68^Ga-Pentixafor could also be potentially employed for targeted and timed therapy to initiate CXCR4 inhibition at the maximum of target expression.

Our study has various limitations, including its retrospective nature, its restriction to a single center, and the small sample size, thus the limiting statistical power. While there is a clinical follow up, no follow up CXCR4 PET/CT was available in this retrospective cohort. A lesion-to-lesion analysis, preferably in a longitudinal setting on ^68^Ga-Pentixafor positive/negative lesions, was beyond the scope of the present study, but it is definitely warranted in further clinical trials. Furthermore, although histology could prove the presence of CXCR4 expression on cells in most of the biopsy specimens, receptor expression was relatively heterogeneous and it could not always be correlated with findings of PET imaging. Histology results also might be influenced by biopsy yields and by receptor kinetics and internalization, given that CXCR4 expression at the cell surface is dynamically regulated and receptor internalization is induced by ligand binding [26]. In addition, biopsies of the primary were not always feasible, such as in subjects diagnosed with CUP (patients #6, #11). Moreover, novel radioligands, such as fibroblast activation protein-targeting compounds, may also allow for further insights into pathophysiology of high grade NET/NEC relative to the reference standard ^18^F-FDG [27].

## 5. Conclusions

Non-invasive imaging of CXCR4 expression in poorly differentiated NEC with ^68^Ga-Pentixafor PET/CT is inferior to ^18^F-FDG PET/CT. In addition, the lesion-based heterogeneity of FDG-avid and Pentixafor-negative lesions, and vice versa, should be further explored in terms of outcome prediction.

## Figures and Tables

**Table 1:** Patient characteristics. All subjects were treatment-naive at timepoint of imaging/biopsy. * at any site; ^†^ IHC scoring system: 0 = negative, 1 = weak, 2 = moderate, 3 = strong; Age in years; CUP, cancer of unknown primary; CXCR4, CXC-motif chemokine receptor 4; ECOG, Eastern Cooperative Oncology Group; F, female; FDG, fluorodeoxyglucose; IHC, immunohistochemistry; Ki-67, proliferation index in [%]; N1, Nodal; LN, lymph node; M1, Metastasis; M, male and PET, positron emission tomography.

				Location of Primary/Metastases			PET-Positive *				IHC ^†^
Case	Sex	Age	ECOG	Primary	N1	M1	FDG	CXCR4	Site of Biopsy	Ki-67	CXCR4
#1	M	56	0	oesophagus	local + distant	liver, lung, bone	yes	yes	oesophagus	60	1
#2	M	76	0	ileum	local	liver	yes	no	liver	70	1
#3	M	70	1	pancreas	local	liver	yes	yes	pancreas	90	3
#4	F	54	1	oesophagus	local	liver, lung	yes	yes	liver	90	2
#5	F	44	0	rectum	none	liver	yes	yes	liver	90	1
#6	M	78	0	CUP	local	none	yes	yes	axillary LN	70	1
#7	M	69	1	stomach	local	liver	yes	yes	stomach	90	1
#8	M	77	0	stomach	distant	liver, lung	yes	yes	stomach	90	2
#9	M	64	1	pancreas	none	liver, stomach	yes	yes	stomach	45	1
#10	M	76	0	stomach	local	liver	yes	yes	stomach	80	1
#11	M	45	0	CUP	local + distant	lung, bone	yes	yes	inguinal LN	50	N/A

**Table 2:** PET results. Displayed data are the respective measurements of the maximum standardized uptake value (SUV_max_). ^18^F-FDG, ^18^F-fluorodeoxyglucose; Lesions^+^, detected lesions and total lesions; M1, hottest haematogenous metastases; N/A, data not available; N1, hottest lymph node metastases; SD, standard deviation and TBR, tumor-to-background ratio of the primary tumor (* SUV_max_ of the primary divided by SUV_mean_ of the background).

	^18^F-FDG					^68^Ga-Pentixafor				
	SUV_max_					SUV_max_				
Case	Primary	TBR_Primary_ *	N1	M1	Lesions^+^	Primary	TBR_Primary_ *	N1	M1	Lesions^+^
#1	17.2	8.9	10.1	20.7	6/6	8.4	5.2	9.3	10.5	5/6
#2	21.3	19.7	6.9	37.1	7/9	N/A	N/A	N/A	N/A	0/9
#3	6.5	4.5	N/A	3.4	8/8	10.2	5.9	9.7	N/A	4/8
#4	15.4	8.3	17.5	N/A	16/17	6.8	4.2	3.7	N/A	5/17
#5	6.5	3.6	2.8	5.0	5/5	3.7	2.9	4.3	3.6	3/5
#6	N/A	N/A	9.2	N/A	2/2	N/A	N/A	11.3	N/A	2/2
#7	9.2	4.7	4.3	11.5	16/16	8.7	5.2	8.3	15.9	3/16
#8	6.7	3.7	6.9	40.5	13/13	5.7	3.6	8.9	14.6	6/13
#9	31.9	22.6	N/A	15.7	7/7	10.6	5.7	N/A	5.8	3/7
#10	5.2	2.8	6.6	13.7	10/12	7.4	5.2	10.2	14.3	7/12
#11	N/A	N/A	21.3	9.6	12/12	N/A	N/A	7.8	9.1	4/12
Mean ± SD	13.3 ± 8.5	8.8 ± 6.9	9.5 ± 5.8	17.5 ± 12.4		7.7 ± 2.2	4.7 ± 1.0	8.2 ± 2.4	11.7 ± 3.5	

## Data Availability

Data is available upon request from the authors.

## References

[1] Klimstra D.S., Modlin I.R., Coppola D., Lloyd R.V., Suster S. (2010). The pathologic classification of neuroendocrine tumors: A review of nomenclature, grading, and staging systems. Pancreas.

[2] Delle Fave G., Sundin A., Taal B., Ferolla P., Ramage J.K., Ferone D., Ito T., Weber W., Zheng-Pei Z., De Herder W.W. (2016). ENETS Consensus Guidelines Update for Gastroduodenal Neuroendocrine Neoplasms. Neuroendocrinology.

[3] Strosberg J., Wolin E., Chasen B., Kulke M., Bushnell D., Caplin M., Baum R.P., Kunz P., Hobday T., Hendifar A. (2018). Health-Related Quality of Life in Patients With Progressive Midgut Neuroendocrine Tumors Treated With (177)Lu-Dotatate in the Phase III NETTER-1 Trial. J. Clin. Oncol..

[4] Baum R.P., Kluge A.W., Kulkarni H., Schorr-Neufing U., Niepsch K., Bitterlich N., Van Echteld C.J. (2016). [(177)Lu-DOTA](0)-D-Phe(1)-Tyr(3)-Octreotide ((177)Lu-DOTATOC) For Peptide Receptor Radiotherapy in Patients with Advanced Neuroendocrine Tumours: A Phase-II Study. Theranostics.

[5] Strosberg J., El-Haddad G., Wolin E., Hendifar A., Yao J., Chasen B., Mittra E., Kunz P.L., Kulke M.H., Jacene H. (2017). Phase 3 Trial of 177Lu-Dotatate for Midgut Neuroendocrine Tumors. N. Engl. J. Med..

[6] Pavel M., Öberg K., Falconi M., Krenning E., Sundin A., Perren A., Berruti A. (2020). Gastroenteropancreatic neuroendocrine neoplasms: ESMO Clinical Practice Guidelines for diagnosis, treatment and follow-up. Ann. Oncol..

[7] Carlsen E.A., Fazio N., Granberg D., Grozinsky-Glasberg S., Ahmadzadehfar H., Grana C.M., Zandee W.T., Cwikla J., Walter M.A., Oturai P.S. (2019). Peptide receptor radionuclide therapy in gastroenteropancreatic NEN G3: A multicenter cohort study. Endocr. Relat. Cancer.

[8] Zhang J., Kulkarni H.R., Singh A., Niepsch K., Müller D., Baum R.P. (2019). Peptide Receptor Radionuclide Therapy in Grade 3 Neuroendocrine Neoplasms: Safety and Survival Analysis in 69 Patients. J. Nucl. Med..

[9] Thang S.P., Lung M.S., Kong G., Hofman M.S., Callahan J., Michael M., Hicks R.J. (2018). Peptide receptor radionuclide therapy (PRRT) in European Neuroendocrine Tumour Society (ENETS) grade 3 (G3) neuroendocrine neoplasia (NEN)—A single-institution retrospective analysis. Eur. J. Nucl. Med. Mol. Imaging.

[10] Rinke A., Gress T.M. (2017). Neuroendocrine Cancer, Therapeutic Strategies in G3 Cancers. Digestion.

[11] Chan D.L., Pavlakis N., Schembri G.P., Bernard E.J., Hsiao E., Hayes A., Barnes T., Diakos C., Khasraw M., Samra J. (2017). Dual Somatostatin Receptor/FDG PET/CT Imaging in Metastatic Neuroendocrine Tumours: Proposal for a Novel Grading Scheme with Prognostic Significance. Theranostics.

[12] Kaemmerer D., Träger T., Hoffmeister M., Sipos B., Hommann M., Sänger J., Schulz S., Lupp A. (2015). Inverse expression of somatostatin and CXCR4 chemokine receptors in gastroenteropancreatic neuroendocrine neoplasms of different malignancy. Oncotarget.

[13] Werner R.A., Weich A., Higuchi T., Schmid J.S., Schirbel A., Lassmann M., Wild V., Rudelius M., Kudlich T., Herrmann K. (2017). Imaging of Chemokine Receptor 4 Expression in Neuroendocrine Tumors—A Triple Tracer Comparative Approach. Theranostics.

[14] Herrmann K., Schottelius M., Lapa C., Osl T., Poschenrieder A., Hänscheid H., Lückerath K., Schreder M., Bluemel C., Knott M. (2016). First-in-Human Experience of CXCR4-Directed Endoradiotherapy with 177Lu- and 90Y-Labeled Pentixather in Advanced-Stage Multiple Myeloma with Extensive Intra- and Extramedullary Disease. J. Nucl. Med..

[15] Lapa C., Herrmann K., Schirbel A., Hänscheid H., Lückerath K., Schottelius M., Kircher M., Werner R.A., Schreder M., Samnick S. (2017). CXCR4-directed endoradiotherapy induces high response rates in extramedullary relapsed Multiple Myeloma. Theranostics.

[16] Lapa C., Hänscheid H., Kircher M., Schirbel A., Wunderlich G., Werner R.A., Samnick S., Kotzerke J., Einsele H., Buck A.K. (2019). Feasibility of CXCR4-Directed Radioligand Therapy in Advanced Diffuse Large B-Cell Lymphoma. J. Nucl. Med..

[17] Martin R., Jüttler S., Müller M., Wester H.J. (2014). Cationic eluate pretreatment for automated synthesis of [⁶⁸Ga]CPCR4.2. Nucl. Med. Biol..

[18] Vag T., Gerngross C., Herhaus P., Eiber M., Philipp-Abbrederis K., Graner F.-P., Ettl J., Keller U., Wester H.-J., Schwaiger M. (2016). First Experience with Chemokine Receptor CXCR4-Targeted PET Imaging of Patients with Solid Cancers. J. Nucl. Med..

[19] Werner R.A., Kircher S., Higuchi T., Kircher M., Schirbel A., Wester H.-J., Buck A.K., Pomper M.G., Rowe S.P., Lapa C. (2019). CXCR4-Directed Imaging in Solid Tumors. Front. Oncol..

[20] Maurer S., Herhaus P., Lippenmeyer R., Hänscheid H., Kircher M., Schirbel A., Maurer H.C., Buck A.K., Wester H.-J., Einsele H. (2019). Side Effects of CXC-Chemokine Receptor 4-Directed Endoradiotherapy with Pentixather Before Hematopoietic Stem Cell Transplantation. J. Nucl. Med..

[21] Linde P., Baues C., Wegen S., Trommer M., Quaas A., Rosenbrock J., Celik E., Marnitz S., Bruns C.J., Fischer T. (2021). Pentixafor PET/CT for imaging of chemokine receptor 4 expression in esophageal cancer—A first clinical approach. Cancer Imaging.

[22] Breun M., Monoranu C.M., Kessler A.F., Matthies C., Löhr M., Hagemann C., Schirbel A., Rowe S.P., Pomper M.G., Buck A.K. (2019). [(68)Ga]-Pentixafor PET/CT for CXCR4-Mediated Imaging of Vestibular Schwannomas. Front. Oncol..

[23] Osl T., Schmidt A., Schwaiger M., Schottelius M., Wester H.J. (2020). A new class of PentixaFor- and PentixaTher-based theranostic agents with enhanced CXCR4-targeting efficiency. Theranostics.

[24] Werner R.A., Thackeray J.T., Diekmann J., Weiberg D., Bauersachs J., Bengel F.M. (2020). The Changing Face of Nuclear Cardiology: Guiding Cardiovascular Care Toward Molecular Medicine. J. Nucl. Med..

[25] Circelli L., Sciammarella C., Guadagno E., Tafuto S., de Caro M.D.B., Botti G., Pezzullo L., Aria M., Ramundo V., Tatangelo F. (2016). CXCR4/CXCL12/CXCR7 axis is functional in neuroendocrine tumors and signals on mTOR. Oncotarget.

[26] Lapa C., Lückerath K., Kircher S., Hänscheid H., Grigoleit G.U., Rosenwald A., Stolzenburg A., Kropf S., Einsele H., Wester H.J. (2019). Potential influence of concomitant chemotherapy on CXCR4 expression in receptor directed endoradiotherapy. Br. J. Haematol..

[27] Komek H., Gundogan C., Can C. (2020). 68Ga-FAPI PET/CT Versus 68Ga-DOTATATE PET/CT in the Evaluation of a Patient With Neuroendocrine Tumor. Clin. Nucl. Med..

